# Quantitation of a Novel Engineered Anti-infective Host Defense Peptide, ARV-1502: Pharmacokinetic Study of Different Doses in Rats and Dogs

**DOI:** 10.3389/fchem.2019.00753

**Published:** 2019-11-13

**Authors:** Alexandra Brakel, Daniela Volke, Carl N. Kraus, Laszlo Otvos, Ralf Hoffmann

**Affiliations:** ^1^Faculty of Chemistry and Mineralogy, Institute of Bioanalytical Chemistry, Universität Leipzig, Leipzig, Germany; ^2^Center for Biotechnology and Biomedicine, Universität Leipzig, Leipzig, Germany; ^3^Arrevus, Inc., Raleigh, NC, United States; ^4^Institute of Medical Microbiology, Semmelweis University, Budapest, Hungary

**Keywords:** antimicrobial peptide (AMP), Beagle dogs, Chex1-Arg20 amide, parallel reaction monitoring (PRM), pharmacokinetics, proline-rich AMP, Sprague-Dawley rats

## Abstract

The designer proline-rich antimicrobial peptide (PrAMP) Chex1-Arg20 amide (ARV-1502) is active against Gram-negative and Gram-positive pathogens in different murine infection models when administered parenterally and possesses a wide therapeutic index. Here we studied the pharmacokinetics of ARV-1502 for the first time when administered intramuscularly or intravenously (IV) in Sprague Dawley rats and Beagle dogs. First, a specific and robust quantitation method relying on parallel reaction monitoring (PRM) using a high-resolution hybrid quadrupole-Orbitrap mass spectrometer coupled on-line to reversed-phase uHPLC was established and validated. The limit of detection was 2 ng/mL and the limit of quantitation was 4 ng/mL when spiked to pooled rat and dog plasma. When ARV-1502 was administered IV at doses of 75 and 250 μg/kg in dogs and rats, the plasma concentrations were 0.7 and 3.4 μg/mL 2 min post-administration, respectively. ARV-1502 plasma concentrations declined exponentially reaching levels between 2 and 4 ng/mL after 2 h. Intramuscular administration of 0.75 mg/kg in dogs and 2.5 mg/kg in rats resulted in a different pharmacokinetics profile. The plasma concentrations peaked at 15 min post-injection at 1 μg/mL (dogs) and 12 μg/mL (rats) and decreased exponentially within 3 h to 4 and 16 ng/mL, respectively. The initial plasma concentrations of ARV-1502 and the decay timing afterwards indicated that the peptide circulated in the blood stream for several hours, at some point above the minimal inhibitory concentration against multidrug-resistant Enterobacteriaceae, with blood concentrations sufficient to suppress bacterial growth and to modulate the immune system.

## Introduction

The rapid global spread of multi-drug resistant (MDR) bacterial pathogens has resulted in the United Nations stating that MDR is one of the greatest threats to Global Health that currently exists (Sprenger, 2016). Worldwide research efforts are devoted to identifying new chemical entities with novel mechanisms against this global threat. One promising class of compounds are antimicrobial peptides (AMPs), defined here as naturally occurring gene-encoded peptides or proteins, that are expressed in all kingdoms of life as part of innate immunity (Haney et al., 2017; Wu et al., 2018). Among these peptides, proline-rich AMPs (PrAMPs) have been intensively studied in recent years (Otvos, 2003; Li et al., 2014) providing interesting lead compounds based on apidaecin (Czihal et al., 2012; Berthold et al., 2013), oncocin (Knappe et al., 2010a,b), and bactenicin (Benincasa et al., 2004) or artificially designed PrAMPs like A3-APO and Chex1-Arg20 amide (recently termed ARV-1502; Chex-RPDKPRPYLPRPRPPRPVR-NH_2_; Chex denotes 1-amino-cyclohexane carboxylic acid), which were engineered as bacterial chaperone protein inhibitors (Otvos et al., 2005; Noto et al., 2008). While the activity spectrum is narrow, ARV-1502 shows good antibacterial activities against Enterobacteriaceae *in vitro*, but remains inactive against Gram-positives, i.e., minimal inhibitory concentrations (MICs) ranging from 4 to 128 μg/mL. Remarkably, the peptide exhibits beneficial *in vivo* efficacies in different murine infection models and pathogens regardless of the phylogenic status of the pathogens (Otvos et al., 2014, 2018; Ostorhazi et al., 2017; Xiong et al., 2019). The unexpectedly potent activities noted *in vivo* were proposed to be linked to immunomodulatory effects of ARV-1502 (Ostorhazi et al., 2011, 2018), although non-optimized MIC-testing conditions or synergistic effects with host-borne substances hypothesized for other PrAMPs may also contribute (Knappe et al., 2016). Nevertheless, ARV-1502 shows a promising clinical profile in murine infection models and low adverse effects in mice. To obtain a better understanding of the *in vivo* characteristics of ARV-1502, we studied its pharmacokinetics in Sprague Dawley rats and Beagle dogs after intramuscular (IM) and intravenous (IV) administration at sub-therapeutic doses.

## Materials and Methods

### Peptide

ARV-1502 was obtained from PolyPeptide Laboratories (San Diego, CA, USA) as (white powder) with a purity of 97.3% according to RP-HPLC (Noto et al., 2008). The residual TFA content was 0.05% (Supplement Certificate). The monoisotopic mass (2473.5 g/mol) was confirmed by ESI-MS providing a mass of 2473.9 after deconvolution. The identity was further confirmed by amino acid analysis (Asx, Pro, Val, Leu, Tyr, Lys, and Arg) (Supplement Certificate). Onc72 was synthesized in-house (VDKPPYLPRPRPPROIYNO-NH_2_, where O represents L-ornithine). Purities and identities of Onc72 and ARV-1502 were analyzed in-house using RP-HPLC, ESI-MS, and ESI-MS/MS (Figure S1).

### Animal Study

The objective of the study conducted at ITR Laboratories Canada Inc (Baie d'Urfe, Quebec, Canada) was to determine the pharmacokinetic profile of ARV-1502 following single intravenous or intramuscular injection administration to Sprague-Dawley rats and Beagle dogs. The plan for this study was reviewed and assessed by the Animal Care Committee (ACC) of ITR. ACC acceptance of the study plan is maintained on file at ITR. All animals used in this study were cared for in accordance with the principles outlined in the current “Guide to the Care and Use of Experimental Animals” as published by the Canadian Council on Animal Care and the “Guide for the Care and Use of Laboratory Animals” published by NIH. For the rat study (Table 1), ARV-1502 was administered to two groups of rats at a dose volume of 0.8 mL/kg (divide equally over 2 sites) and 1.0 mL/kg for IM and IV routes, respectively.

**Table 1:** ARV-1502 pharmacokinetic study in Sprague-Dawley rats.

**Group**	**Treatment**	**Dose level (mg/kg)**	**Dose concentration (mg/mL)**	**Number of animals**	
				**Male (BW)**	**Female (BW)**
1	IM	2.5	3.125	6 (279 ± 5.8 g)	6 (211 ± 5.9 g)
2	IV	0.25	0.25	6 (273 ± 8.5 g)	6 (213 ± 6.0 g)

*BW denotes body weight*.

A series of eight blood samples (~0.5 mL each) was collected in rats on Day 1. Following IM administration, the blood was collected at 5, 15, 30, and 45 min and at 1, 1.5, 2, and 3-h post-dose. After IV administration, blood was collected at 2, 5, 15, 30, and 45 min and at 1, 1.5, and 2-h post-dose. Animals were euthanized after the last blood collection by carbon dioxide asphyxiation followed by cervical dislocation and discarded without further examination. Safety parameters monitored on the study were mortality and clinical signs. Body weights (BW) were recorded to calculate the dose only. Blood sampling was performed for pharmacokinetic evaluation.

For the dog study, ARV-1502 was administered once IM (divided equally over 2 sites) and once IV (1 week apart) as detailed in Table 2. Following dosing, blood was collected at the same time points noted above for the rat study. Dogs were released from the study after blood sampling. Safety parameters monitored on the study were mortality and clinical signs.

**Table 2:** ARV-1502 pharmacokinetic study in Beagle dogs.

**Study day**	**Treatment**	**Dose level (mg/kg)**	**Dose concentration (mg/mL)**	**Dose volume (mL/kg)**	**Number of animals (BW)**
1	IM	0.75	1.5	0.5	3 (10.2 ± 0.47 kg)
8	IV	0.075	0.15	0.5	3

*BW denotes body weight*.

### Sample Preparation

Aliquots of rat or Beagle plasma (20 μL) were spiked with standard peptide Onc72 (0.35 μg/mL plasma, 1.2 μL). Samples were diluted with aqueous TFA in two steps (0.2% v/v, 20 μL and 0.1% v/v, 260 μL) and loaded on an Oasis HLB 96-well-SPE-plate (5 mg sorbent per well, Waters). The samples were washed twice with aqueous TFA (0.1% v/v, 1 mL) and eluted in two steps with aqueous acetonitrile (30% v/v, 150 μL) containing formic acid (0.1% v/v). The fractions were dried in a vacuum centrifuge (SpeedVac, 60°C), reconstituted with aqueous methanol (75% v/v, 20 μL) containing formic acid (0.1% v/v), and diluted with aqueous acetonitrile (3% v/v, 100 μL) containing formic acid (0.1%).

### RP-uHPLC-MS/MS

A quadrupole-Orbitrap mass spectrometer (Q Exactive plus™, Thermo Scientific) was coupled online to an uHPLC (Vanquish™, Thermo Scientific). Separation was achieved on a Jupiter C_18_ column (inner diameter: 1 mm, length: 150 mm, particle size: 5 μm, pore size: 130 Å, Phenomenex Ltd.) at 55°C using a flow rate of 200 μL/min and a linear gradient of 3 to 23% (v/v) aqueous acetonitrile containing formic acid (0.1%) within 5 min.

ARV-1502 was quantitated by the peak area of an extracted ion chromatogram (XIC) obtained from the MS/MS using *Xcalibur 4.0.27.21* software. The peak area of ARV-1502 corrected by using the peak area of Onc72. Pharmacokinetic datasets were evaluated with a PKSolver add-in for Microsoft Excel using a non-compartmental analysis (Zhang et al., 2010).

## Results

Recent reports on the pharmacokinetics of other PrAMPs in mice (Holfeld et al., 2015; Schmidt et al., 2016, 2017) indicated rapid clearance rates after intraperitoneal and intravenous injections with elimination half-life times ranging from 15 to 30 min. Assuming similar pharmacokinetic properties of ARV-1502, an LC-MS analytics was developed to quantify ARV-1502 spiked to pooled rat and Beagle plasma at concentrations below 10 ng/mL. Due to the high content of Arg- and Lys-residues in ARV-1502 and Onc72 (a control PrAMP that was used as internal standard), electrospray ionization (ESI) yielded high charge states with the signals corresponding to [M+6H]^6+^-ions being the most intense. The transitions selected relied on intense fragment ions displaying higher *m/z*-values than the 6-fold protonated precursor ion, i.e., triply or quadruply protonated y-ions (Table 3). As the isotope patterns of such charge states are not resolved in a quadrupole mass analyzer, the method was developed on an Orbitrap analyzer that also provides increased mass accuracy. The parallel reaction monitoring (PRM) offers the additional advantage that all fragment ions are recorded allowing reconsidering quantifiers and qualifiers after acquiring all spectra. While peptide quantitation by PRM is well-established, the method development was hampered by the physicochemical properties of ARV-1502. As reported for Onc72 (Schmidt et al., 2016), ARV-1502 was also gradually lost in glass inserts before injected by the autosampler despite acidic conditions [0.1% (v/v) formic acid] and low temperatures (10°C). In contrast to Onc72 that did not adsorb to polypropylene inserts, ARV-1502 was mostly lost when stored in acidic solutions for 10 h at 10°C (Figure S2). The presumed hydrophobic binding was prevented by using higher organic contents, such as 12.5% (v/v) methanol or acetonitrile. The established analytics relied on solid phase extraction (SPE) in a 96-well format to remove most plasma components, e.g., salts, proteins, and lipids, and quantitation by LC-MS (Table 3, Table S1 and Figure S3) with the quantifier being the remaining precursor ion in the tandem mass spectrum due to the higher sensitivity (confirmed for quantities above 37 ng/mL by qualifier I). Finally, limits of detection (LOD) and quantitation (LOQ) of 2 and 4 ng/mL, respectively, and a linear dynamic range (LDR) from 15 to 3.7 μg/mL (*R*^2^ = 0.9981) were achieved in spiked rat and Beagle plasma. This appeared to be sufficiently sensitive for the intended pharmacokinetic study, especially as ARV-1502 concentrations below the LDR could be quantitated by an exponential fit. As Onc72 eluted only 15 s later than ARV-1502, it was added as internal standard to correct the peak areas of ARV-1502, i.e., to compensate peptide losses and pipetting errors especially at low peptide concentrations. Recovery rates of ARV-1502 were 56 ± 10% after sample preparation. Intraday and interday precisions were 7% or better for concentrations above 1.5 μg/mL and up to 20% at lower concentrations. Details of the optimized method are provided as Tables S1, S2.

**Table 3:** Transitions selected for the quantitation of ARV-1502 and internal standard Onc72 and the respective normalized collision energies (NCE).

**Peptide**	Type^*^	**Precursor**			**Fragment**			**NCE**
		**Ion**	***m/z*_**theo**_**	***m/z*_**exp**_**	**Ion**	***m/z*_**theo**_**	***m/z*_**exp**_**	
ARV-1502	Quantifier I	[M+6H]^6+^	413.421	413.421	[M+6H]^6+^	413.421	413.421	18%
	Qualifier I	[M+6H]^6+^	413.421	413.421	y_15_^4+^	464.037	464.034	23%
	Qualifier II	[M+5H]^5+^	495.904	495.904	y_18_^4+^	549.080	549.081	28%
	Qualifier III	[M+6H]^6+^	413.421	413.421	y_13_^3+^	533.995	533.993	23%
Onc72 (IS)	Quantifier	[M+6H]^6+^	385.063	385.062	y_15_^3+^ - H_2_O	617.028	617.029	24%
	Qualifier I	[M+6H]^6+^	385.063	385.062	y_16_^4+^ - NH_3_	487.591	487.537	24%
	Qualifier II	[M+6H]^6+^	385.063	385.062	y_15_^3+^	623.080	623.039	24%

* *Note: Qualifier I was used as quantifier II for ARV-1502 quantities above 37 ng/mL to confirm the quantitation of quantifier I*.

**Figure 1:**
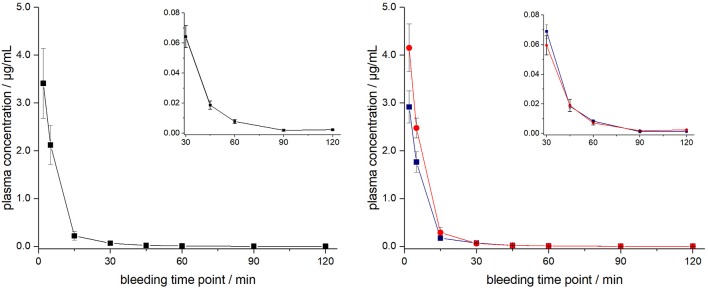
Plasma profile of ARV-1502 after intravenous administration in six male and six female rats at doses of 0.25 mg/kg body weight. Blood samples were collected in three animals of each sex after 2, 15, 45, and 90 min and in the other six rats after 5, 30, 60, and 120 min. Shown are the mean plasma concentrations of six rats per time point (left) and the three male (blue) and three female rats (red, right). The small inserts show the profiles from 30 to 120 min.

**Figure 2:**
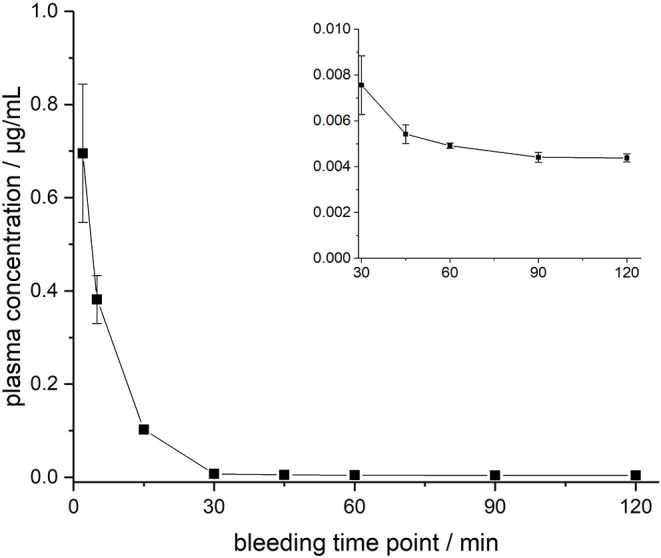
Plasma profile of ARV-1502 after intravenous administration in three male Beagle dogs at doses of 75 μg/kg body weight. Blood samples were collected after 2, 5, 15, 30, 45, 60, 90, and 120 min. The small insert shows the profile from 30 to 120 min.

ARV-1502 injected IV to 12 rats at a dose of 0.25 mg/kg yielded an initial average plasma concentration of 3.1 ± 0.73 μg/mL (blood taken from 6 rats at each time point) 2 min after administration (Figure 1, Figure S4). The plasma concentration decreased in all animals exponentially to 64.2 ± 7.3 ng/mL after 30 min and 2.2 ± 0.4 ng/mL after 2 h with an elimination half-life time (t_1/2_) of 15.4 min. Interestingly, the peptide plasma concentrations were around 25% lower in male rats than in female rats at the first two time points (Figure 1, right panel), but reached similar levels after 45 min. The 70% lower dose administered IV in Beagle (75 μg/kg) was chosen based on earlier toxicity evaluations and also included allometric dose scaling. It provided around five times lower initial plasma levels of 0.69 ± 0.15 μg/mL (2 min; Figure 2, Figure S5). The concentration decreased afterwards with a similar kinetics (t_1/2_ = 7.6 min) as observed for rats reaching peptide concentrations of 7.6 ± 1.3 ng/mL after 30 min and 4.4 ± 0.2 ng/mL after 2 h.

**Figure 3:**
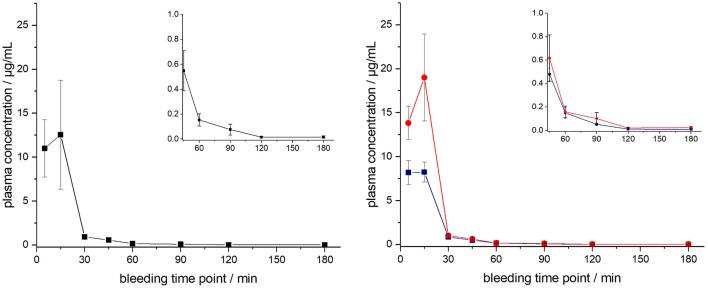
Plasma profile of ARV-1502 after intramuscular administration in six male and six female rats at doses of 2.5 mg/kg body weight. Blood samples were collected in three animals of each sex after 5, 30, 60, and 120 min and in the other six rats after 15, 45, 90, and 180 min. One plasma sample of female rat 1505A collected 15 min post injection was disregarded, as it contained very low peptide quantities (see Figure S6). Shown are the plasma concentrations of all 12 animals (left) and the six male (blue) and six female rats (red, right). The small inserts show the profiles from 45 to 180 min.

**Figure 4:**
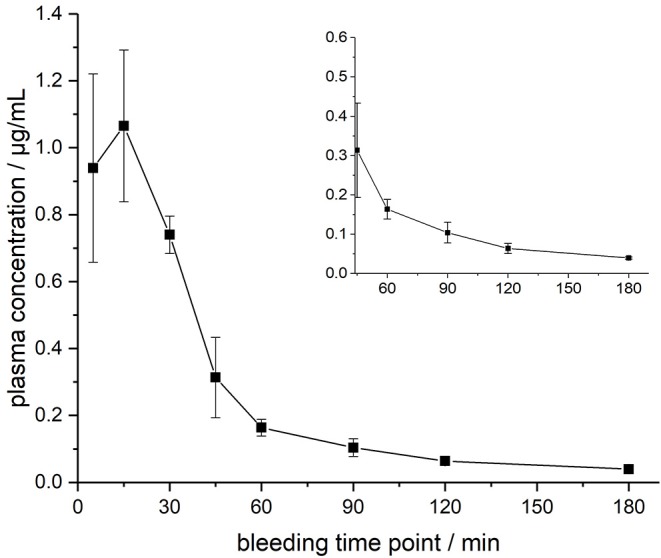
Plasma profile of ARV-1502 after intramuscular administration in three male Beagle dogs at doses of 0.75 mg/kg body weight. Blood samples were collected after 5, 15, 30, 45, 60, 90, 120, and 180 min. The small insert shows the profile from 45 to 180 min.

The ARV-1502 doses used for IM administration were 10 times higher than for IV injection. In rats, ARV-1502 was detected at a concentration of 11.0 ± 3.3 μg/mL after 5 min, which increased slightly within the following 10 min, before it dropped rapidly to around 0.9 ± 0.2 μg/mL after 30 min followed by a slow clearance in the remaining period (Figure 3, Figure S6). Again, the initial peptide levels were around 40% lower in males (8.2 ± 1.4 μg/mL) than in females (13.8 ± 1.9 μg/mL). Interestingly, the concentrations increased in females until the second time point (15 min) to 19.0 ± 4.9 μg/mL, but remained stable in males, before they dropped significantly to a similar level after 30 min (0.8 and 1.0 μg/mL, respectively) followed by an exponential decrease until 3 h post-injection. Beagle (all males) showed a similar profile after IM administration (dose: 0.75 mg/kg) as male rats, i.e., an increase of the ARV-1502 level from 0.94 ± 0.28 μg/mL (5 min) to 1.1 ± 0.2 μg/mL (15 min). Afterwards, the concentration decreased exponentially to a final level of 39.6 ± 2.7 ng/mL after 3 h (Figure 4, Figure S7).

The circulating blood volume (BV) of rats was calculated based on the average body weight (BV = 0.77 + 0.06 × BW) (Lee and Blaufox, 1985), i.e., ~17.5 mL for males, ~13.4 mL for females, and on average ~15.5 mL for all rats. This corresponded well to the volume of distribution, i.e., the theoretical plasma volume containing the total amount of administered ARV-1502, calculated for IV injection (Table 4), which was ~13.1 mL and thus 85% of the theoretical calculation. Intramuscular administration provided a more than 4-fold higher volume confirming the expected slower release from the injection site (depot effect, Table 4). Importantly, the area under the concentration-time curve (AUC_0→∞_) value ratio resembled closely the IM/IV-dose ratio of 10 (Table 4). For dogs the circulating blood volume was assumed to be 86 mL/kg^1^ suggesting an average volume of around 880 mL per animal in the studied dog group. The volumes of distribution calculated for IV injection was 735 mL, i.e., ~84% of BV, and 8.14 L for IM administration, i.e., more than 9-fold larger than the BV in dogs (Table 4). This indicates a stronger depot effect in dogs than in rats.

**Table 4:** Mean pharmacokinetic parameters determined for ARV-1502 in dogs and rats using PKSolver.

	**Dog**		**Rat (male/female)**	
Dose (mg/kg body weight)	0.075	0.75	0.25	2.5
Administration route	Intravenous	Intramuscular	Intravenous	Intramuscular
C_max_ / μg/mL	0.70	1.07	3.41	12.54
C_0_ / μg/mL	1.04	0.94	4.68 (4.1/5.8)	11.00 (8.2/13.8)
t_1/2_ / min	7.6	15.75	15.4	24.2
k / min^−1^	0.09	0.04	0.045	0.029
volume of distribution [mL]	735	8138	13.1 (17.0/9.1)	55.2 (85.1/38.2)
AUC_0 → ∞_/μg·min/mL	8.61	52.73	31.26	296.2
AUMC_0 → ∞_/μg·min^2^/mL	820.3	2729.9	194.6	4248.7
Mean residence time/min	7.07	22.57	6.22	14.34

## Discussion

ARV-1502 is the *in vivo* active metabolite of an engineered dimeric PrAMP that was designed by aligning and combining sequences from nine natural insect-derived PrAMPs, resembling mostly the N-terminal residues of pyrrhocoricin and the C-terminal residues of apidaecin, and replacing the N-terminal residue by amino-cyclohexyl carboxylic acid (Chex; Otvos et al., 2005). ARV-1502 appears to rely on similar mechanisms as described for native PrAMPs. First, its direct bacterial killing identified in broth microdilution assays was linked first to inhibition of DnaK-assisted protein folding (Otvos et al., 2005; Noto et al., 2008) and more recently research on related PrAMPs indicated an inhibition of protein translation in the 70S ribosome (Krizsan et al., 2014, 2015; Graf and Wilson, 2019). Both mechanisms require a certain peptide concentration to kill bacteria or at least to prevent their growth. However, the fast and irreversible bacterial uptake reported for PrAMPs may indicate that the initial peptide concentration, i.e., C_max_ in pharmacokinetic terms, is more important than the time over MIC but likely does not mirror parameters of more traditional antibiotics, such as aminoglycosides. In rats, C_max_ was 3.4 μg/mL IV and 12.54 μg/mL IM after 2 and 15 min, respectively.

The therapeutic index of the dimeric version of the Chex1-Arg20 peptide (A3-APO) is improved when moving from intravenous to intramuscular administration, likely because of the involvement of intramuscular macrophages and providing a depot for slow release into the circulation (Ostorhazi et al., 2017). Indeed, progestin from the contraceptive drug medroxyprogesterone acetate is released slowly from the muscle and has a prolonged duration of action for as long as 3 months (Mishell, 1996).

The depot effect is an important criterion for envisaged human applications of ARV-1502. The increased toxicity signal observed in dogs compared to male rats permits the most conservative estimation of a therapeutic index in humans and will serve as a key factor in estimating a safe starting dose. Such an effect has been well-characterized for other therapeutics, Depo-Provera (depot-medroxyprogesterone acetate, DMPA) being a salient example. Toxicity of DMPA has been studied in mice, rats, rabbits, beagle dogs, and rhesus monkeys after either subcutaneous or intramuscular administration (Jordan, 1994). Different test animal species responded differently to DMPA dosages with only dogs producing tumors. Dose-related effect for the tumor production response in dogs likely reflects the increased drug absorptions compared to other animal species.

Recent rodent experiments in which ARV-1502 was administered to *A. baumannii*-challenged mice demonstrated potent activity at doses of 1.25 mg/kg (Xiong et al., 2019). Allometrically scaled this would be a dose equivalent to 2.5 mg/kg in a rat, which would be 10x that administered in this study. If the C_max_ were proportional, then in those studies the C_max_ would have been ~34 μg/mL, still below the MIC of the *A. baumannii* strain, which was noted to be 50 μg/mL, but certainly sufficient to slow down the bacterial growth rate. It may be that rather than exceeding the MIC, achieving some proportion of the MIC would be sufficient for *in vivo* activity since these peptides have greater than one mode of action and such activity is not fully captured with *in vitro* MIC testing providing ideal bacterial growth conditions. Alternatively, standard MIC testing may simply not be an adequate assay for screening such peptides and alternative methods will need to be employed.

The *in vivo* efficacy might be further explained by the long post-antibiotic effect (PAE), which lasted for 5 to 12 h when *E. coli, K. pneumoniae*, and *P. aeruginosa* were incubated with ARV-1502 at MIC for 1 h and still for at least 3 h at 0.4x MIC (Holfeld et al., 2018). The long PAE indicates a reduced bacterial growth for the mentioned periods allowing the host immune system to clear most pathogens. For example, ARV-1502 cleared pathogens in different murine infection models despite being considered inactive in MIC-testing (MIC > 128 μg/mL) against the same bacterial strains (Ostorhazi et al., 2011, 2018). Considering the pharmacokinetic data presented here, these blood levels will not be reached, even at high therapeutic doses. However, immunomodulatory effects regarding ARV-1502's impact on IL-10 require most likely much lower ARV-1502 concentrations, as a previous study revealed around 2-, 4-, and 5-fold higher IL-10 levels after 24 h when ARV-1502 was injected im at doses of 2, 5, and 10 mg/kg, respectively. Thus, ARV-1502 might activate immune cells for several hours while circulating in the blood stream below the MIC or the pharmacokinetics LOD levels. During this time, the peptide might diffuse into infected tissues or might be released in the tissues from bacteria killed initially by ARV-1502, which can stimulate the immune system at the site of infection for extended periods. The same should be true for dogs, if similar doses are injected. The clearance rate in dogs was around half of the rate in rats, but this might be affected by the four times lower doses applied. At equal doses, the clearance rates might be similar.

## Data Availability Statement

The datasets generated for this study are available on request to the corresponding author.

## Ethics Statement

The animal study was reviewed and approved by Animal Care Committee (ACC) of ITR Laboratories Canada Inc.

## Author Contributions

CK and LO designed the animal studies. RH, AB, and DV designed the experiments to quantify peptides. Mass spectrometric analysis was performed by AB and DV. The manuscript was drafted by RH and the figures were prepared by AB. All authors critically revised the manuscript, approved the final version, and agreed to be accountable for all aspects of the work.

### Conflict of Interest

CK is the Chief Executive Officer of Arrevus, Inc., a biotechnology company developing ARV-1502. LO is the inventor of the composition of matter patent covering peptide ARV-1502. The patent is owned by Temple University and licensed to Arrevus where LO serves as an advisor. RH is cofounder and shareholder of AMP-Therapeutics GmbH (Leipzig, Germany) and a member of the scientific advisory boards of AMP-Therapeutics GmbH and EnBiotix, Inc (Cambridge, USA). The remaining authors declare that the research was conducted in the absence of any commercial or financial relationships that could be construed as a potential conflict of interest.

## Footnotes

^1^A Compendium of Drugs Used for Laboratory Animal Anesthesia, Analgesia, Tranquilization and Restraint. Available online at: https://web.archive.org/web/20110606212907/http://www.drexelmed.,edu/documents/ULAR/IACUC_drugs.pdf (accessed August 18, 2019).
