# Supplementary material for: Quantitation of a Novel Engineered Anti-infective Host Defense Peptide, ARV-1502: Pharmacokinetic Study of Different Doses in Rats and Dogs
Source: Front Chem. 2019 Nov 13;7:753. doi: 10.3389/fchem.2019.00753 (PMC6863955; doi:10.3389/fchem.2019.00753)
Supplement: Supplementary file 1 [file Data_Sheet_1.pdf]

SUPPLEMENT

Pharmacokinetic study of peptide ARV-1502 in male and female rats and male beagles

Alexandra Brakel[1,2], Daniela Volke[1,2], Carl Kraus[3], Laszlo Otvos[3], and Ralf Hoffmann[1,2]

[1]Institute of Bioanalytical Chemistry and [2]Center for Biotechnology and Biomedicine, Universität Leipzig, Leipzig, Germany; [3]Arrevus, Inc., Raleigh, NC, U.S.A.

Table S1: XIC of ARV-1502 quantifier 2

Table S2: Pharmacokinetics in rats after iv injection 2

Certificate of Analysis 3

Figure S1: Characterization of ARV-1502 and Onc72 5

Figure S2: Peptide losses obtained in glass and polypropylene (PP) inserts 6

Figure S3: XIC of ARV-1502 quantifier 7

Figure S4: Pharmacokinetics in rats after iv injection 8

Figure S5: Pharmacokinetics in beagle after iv injection 9

Figure S6: Pharmacokinetics in rats after im injection 10

Figure S7: Pharmacokinetics in beagle after im injection 11

Table S1: Settings applied on uHPLC (VanquishTM, Thermo Scientific) and quadrupole-Orbitrap mass spectrometer (Q Exactive plusTM, Thermo Scientific) for quantification of ARV-1502.

Parameter	Settings
Autosampler temperature	10 °C
Column temperature	55 °C
Flow rate	200 µL/min
Injection volume	100 µL
Wash speed	10 µL/s
Dispense speed	15 µL/s
Draw speed	20 µL/s
Spray voltage	3500 V
Ionization mode	positive
Capillary temperature	300 °C
Sheath gas	40 units
Aux gas	10 units
Probe heater temperature	370 °C

Table S2: Results of validation of the sample preparation and RP-HPLC-PRM method for spiked Beagle plasma. Precision was determined from three replicates on three days.

Peptide	Plasma concentration	Amount on column	Precision		Recovery after SPE
			Intraday	Interday	
ARV-1502	15 ng/mL	0.1 pmol	6-21 %	7 %	ND
	150 ng/mL	1 pmol	6-10 %	14%	59 %
	1.5 µg/mL	10 pmol	3-9 %	7 %	51 %

CERTIFICATE OF ANALYSIS

Revision 00

Name: **ARV-1502**

Lot Number: **AHF32B // 105078-18** Grade: **Research Grade**

Sequence: **H-AChex-Arg-Pro-Asp-Lys-Pro-Arg-Pro-Tyr-Leu-Pro-Arg-Pro-Arg-Pro-Pro-Arg-Pro-Val-Arg-NH2**

Counter Ion: **Acetate**

TEST	METHOD	SPECIFICATIONS	RESULTS
Appearance	Visual inspection	White to off-white powder	White powder
Identity	Mass Spectrometry	M.W. (average) = 2475.0<br>M.W. (monoisotopic) = 2473.5 ± 1 amu	$(M+2H)^{2+}/2$ = 1238.2<br>$(M+3H)^{3+}/3$ = 826.0<br>$(M+4H)^{4+}/4$ = 619.8<br>After deconvolution:<br>M.W. = 2474.9 amu
	Tandem MS	Conforms to the sequence	Conforms to the sequence
	Amino Acid Analysis (AAA)	Asx 0.9 – 1.2<br>Pro 6.8 – 9.2<br>Val 0.9 – 1.2<br>Leu 0.9 – 1.2<br>Tyr 0.9 – 1.2<br>Lys 0.9 – 1.2<br>Arg 5.1 – 6.9<br>Correct Composition ± 15%	1.0<br>8.0<br>1.0<br>1.0<br>1.0<br>1.0<br>6.0
Purity	RP-HPLC (Area normalized)	≥ 95%	97.3%
Related Substances	RP-HPLC (Area normalized)	≤ 5%	2.7%
Acetic Acid Content	RP-HPLC	≤ 20%	16.1%
Net Peptide Content (NPC)	Nitrogen Analysis	≥ 70%	78.4%

PolyPeptide Laboratories San Diego
9395 Cabot Drive - San Diego, CA 92126 - USA
Phone: (858) 408-0808 - Toll Free: (800) 338-4965 - Fax: (858) 408-0799
www.polypeptide.com

PolyPeptide GROUP

CERTIFICATE OF ANALYSIS

Revision 00

Name: **ARV-1502**

Lot Number: **AHF32B // 105078-18** Grade: **Research Grade**

Sequence: **H-AChex-Arg-Pro-Asp-Lys-Pro-Arg-Pro-Tyr-Leu-Pro-Arg-Pro-Arg-Pro-Pro-Arg-Pro-Val-Arg-NH2**

Counter Ion: **Acetate**

TEST	METHOD	SPECIFICATIONS		RESULTS
Water Content	Karl Fischer USP <921>	≤ 10%		6.6%
Residual TFA	RP-HPLC	≤ 0.25%		0.05%
Residual Solvents	GC USP <467>	Acetonitrile	Report result	48 ppm
		Dimethylformamide	Report result	ND (<25 ppm)
		Methanol	Report result	ND (<10 ppm)
		Isopropyl Alcohol	Report result	ND (<10 ppm)
		Methyl tert-butyl ether	Report result	ND (<10 ppm)
		Hexane	Report result	ND (<5 ppm)
		Diisopropyethylamine	Report result	ND (<10 ppm)
		Piperidine	Report result	ND (<100 ppm)
Total Mass Balance	Calculation: NPC (% Nitrogen) + AcOH + H_2O	90 – 105%		101.1%

Date of Manufacture: **January 10, 2018**

Storage: **-20°C ± 5°C**

Revision History:

00 – Initial Release

Comments:

Quality Control by: [signature]

Date: 01/23/18

PolyPeptide Laboratories San Diego
9395 Cabot Drive - San Diego, CA 92126 - USA
Phone: (858) 408-0808 - Toll Free: (800) 338-4965 - Fax: (858) 408-0799
www.polypeptide.com

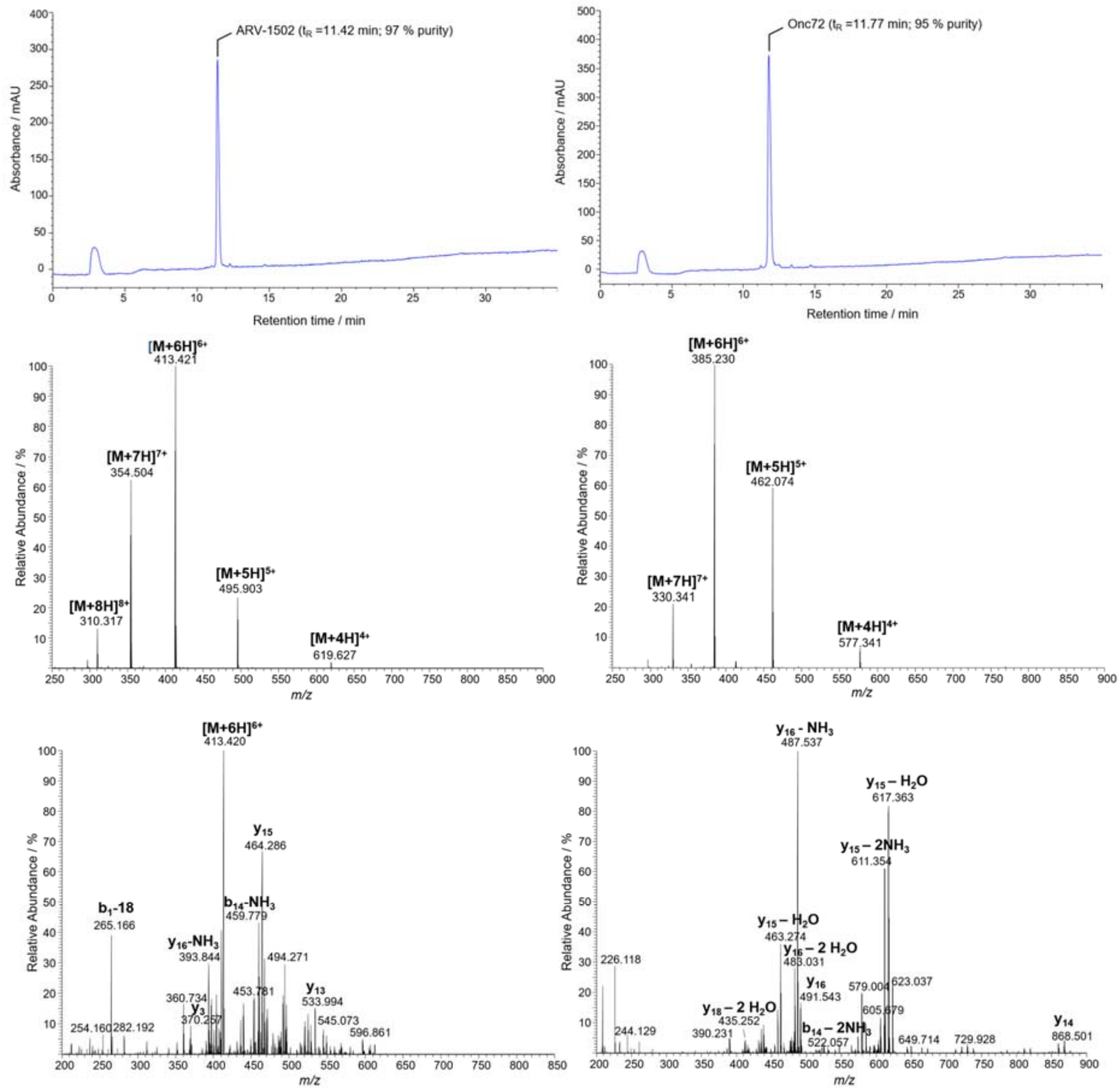

Figure S1: RP-chromatograms (top), ESI-MS (middle), and ESI-MS/MS (bottom) of peptides ARV-1502 (left) and Onc72 (right).

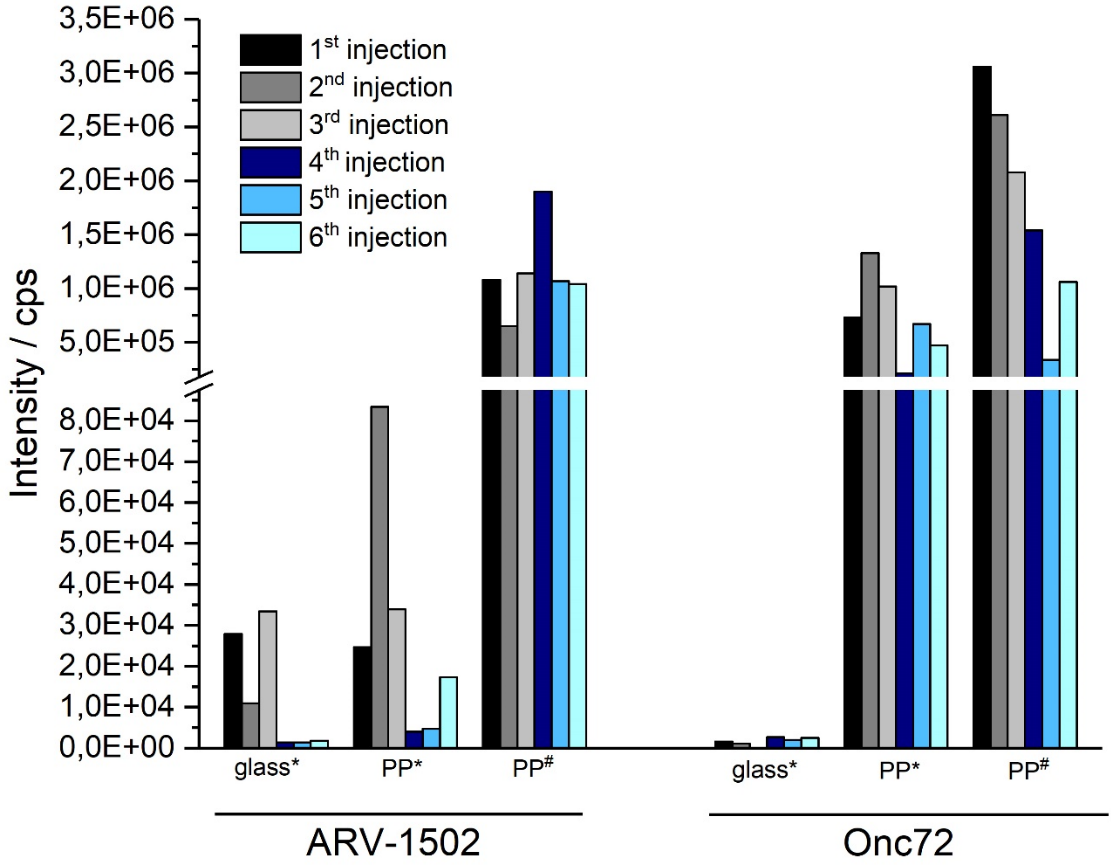

Figure S2: Peptide losses obtained in glass and polypropylene (PP) inserts used in the autosampler. ARV-1502 (left) or Onc72 (right; 0.1 ng/µL) were dissolved in 3 % (v/v) aqueous acetonitrile containing 0.1 % (v/v) formic acid (marked by asterisk) or 12.5 % (v/v) aqueous methanol containing 2.3 % (v/v) acetonitrile and 0.1 % (v/v) formic acid (marked by hashtag). All samples were prepared in parallel and transferred in six glass or six polypropylene inserts at 10 °C, respectively. Samples were injected (10 µL, full-loop injection) to a reversed-phase column (iKey, 150 µm x 50 mm Peptide BEH C18, 130 Å, 1.7 µm, Waters) and were separated with a linear gradient from 5 to 25 % acetonitrile in 5 min (40 °C, 0.3 µL/min using a M-class UPLC coupled to a Synapt G2-Si (Waters). Quantitation relied on the peak intensity of the extracted ion chromatograms. Three samples of each peptide and insert type were analyzed. The remaining samples four to six of each peptide and insert type were analyzed 10 hours later.

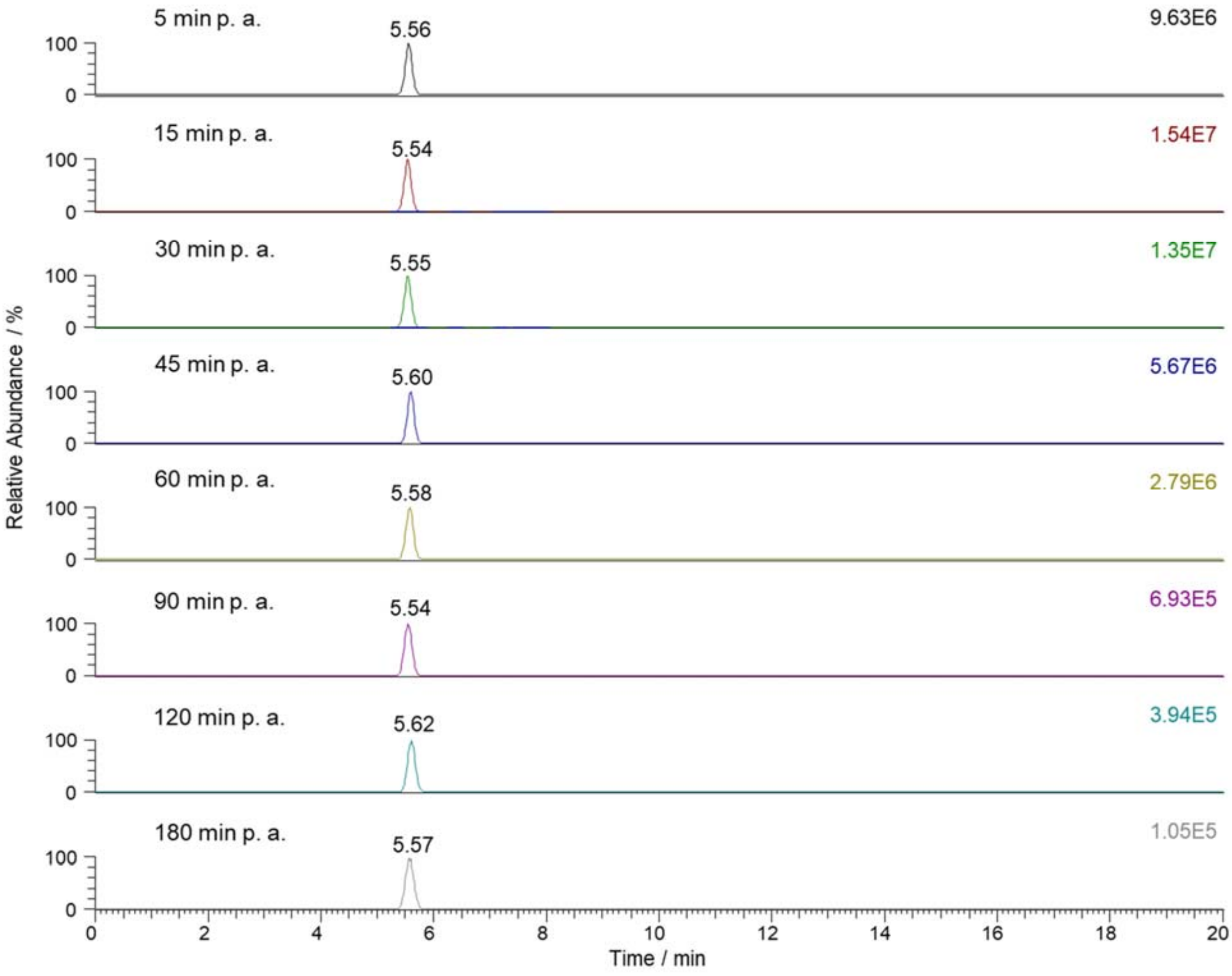

Figure S3: Extracted ion chromatogram (XIC) of the quantifier selected for ARV-1502 in plasma samples obtained from one beagle after intramuscular injection.

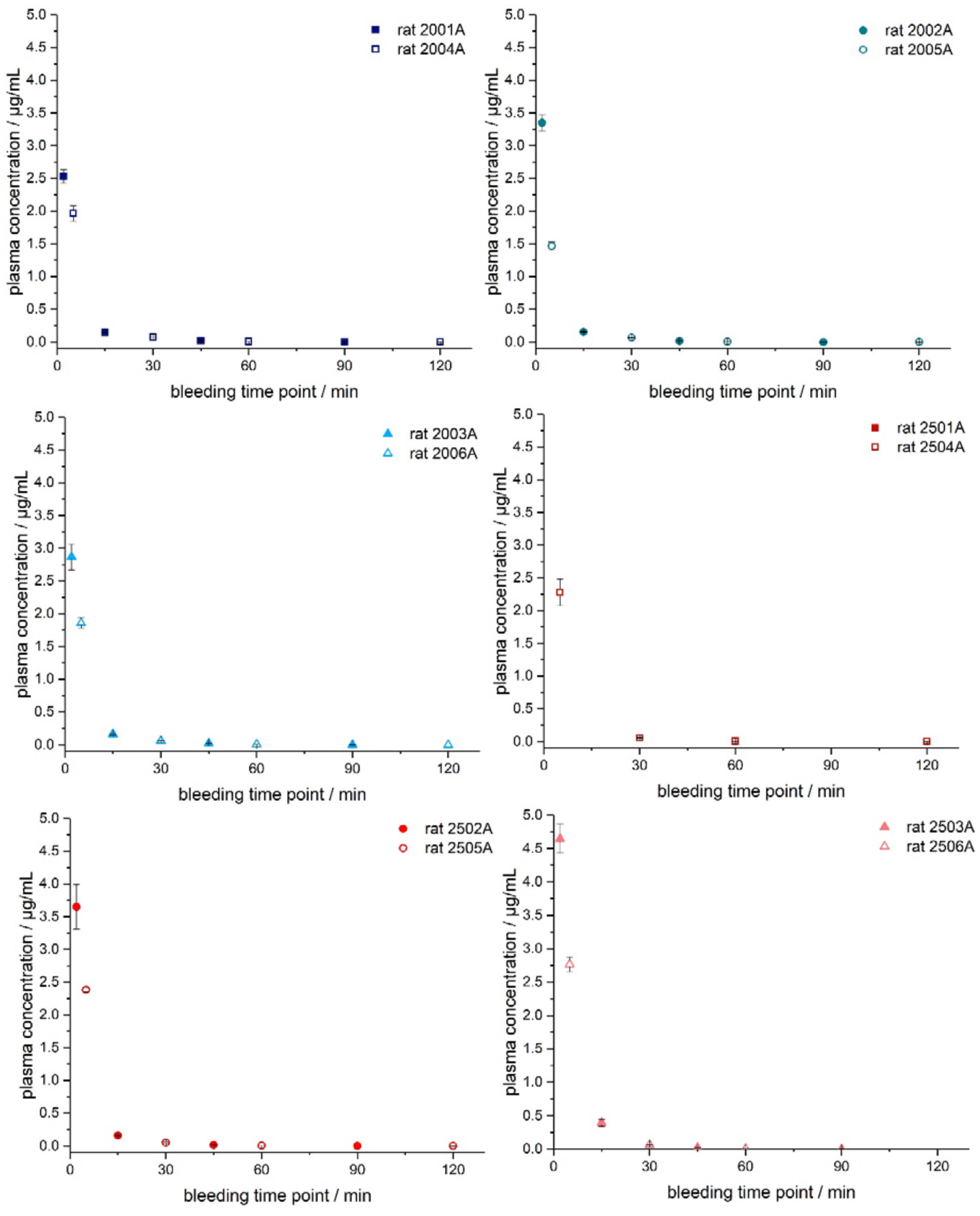

Figure S4: Plasma profile of ARV-1502 after intravenous administration in six males (2001A to 2006A) and six females (2510A to 2506A) at doses of 0.25 mg/kg body weight. Blood samples were collected from each rat for times after either 2, 15, 45, and 90 min or 5, 30, 60, and 120 min.

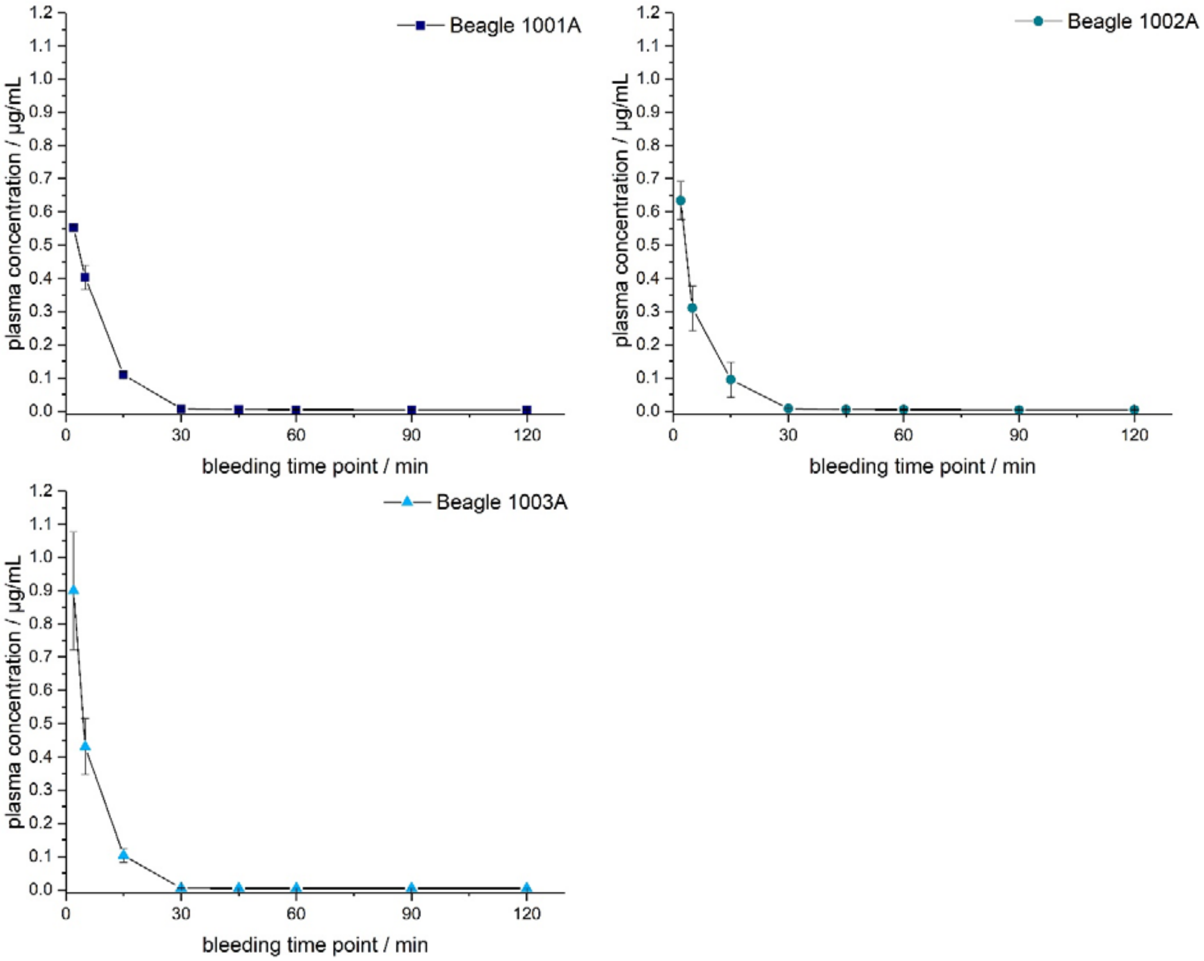

Figure S5: Plasma profile of ARV-1502 after intravenous administration in three male beagle dogs (1001A to 1003A) at doses of 75 µg/kg body weight. Blood samples were collected in each animal after 2, 5, 15, 30, 45, 60, 90, and 120 min.

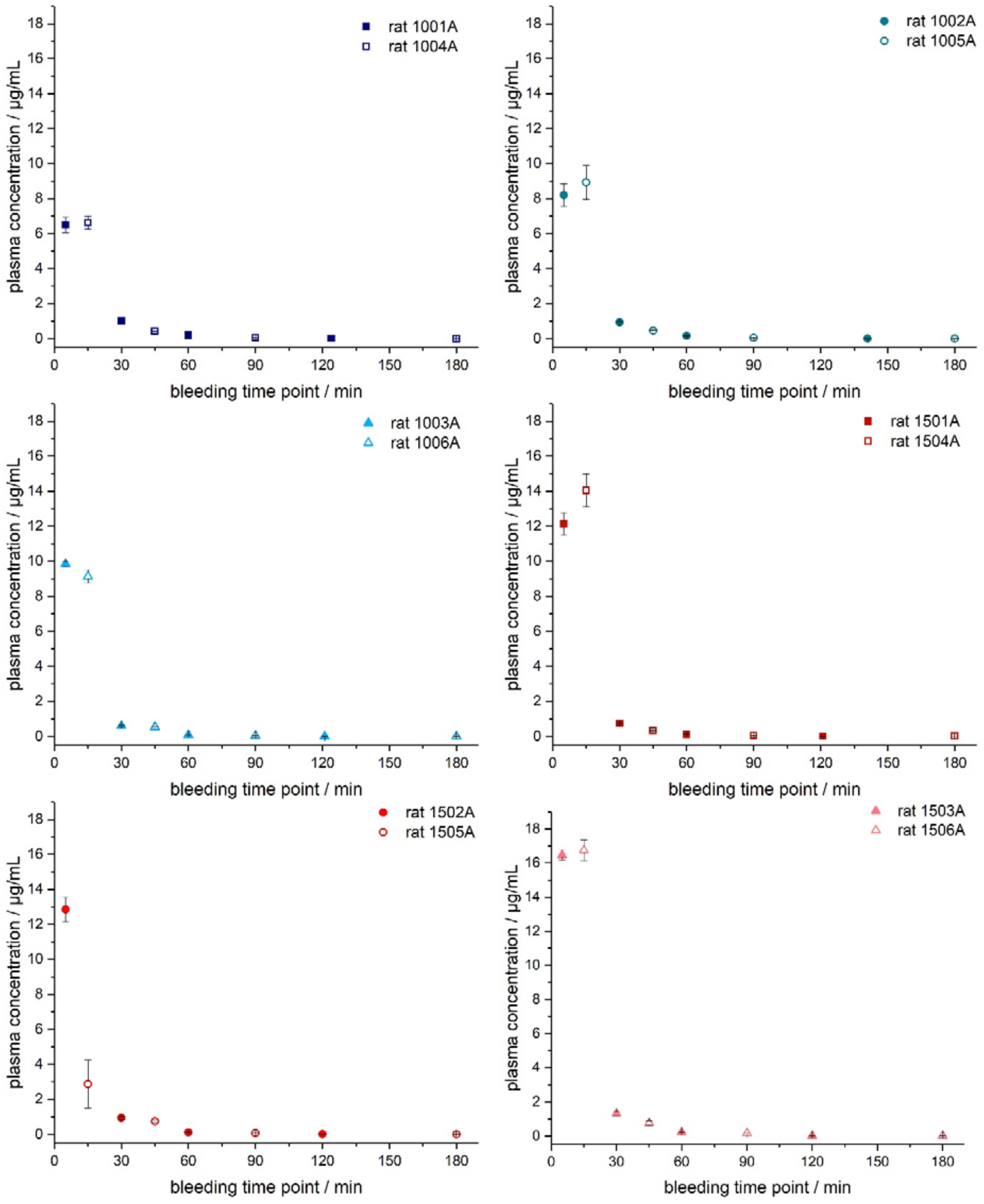

Figure S6: Plasma profile of ARV-1502 after intramuscular administration in six males (1001A to 1006A) and six females (1510A to 1506A) at doses of 2.5 mg/kg body weight. Blood samples were collected from each rat four times after either 5, 30, 60, and 120 min or 15, 45, 90, and 180 min.

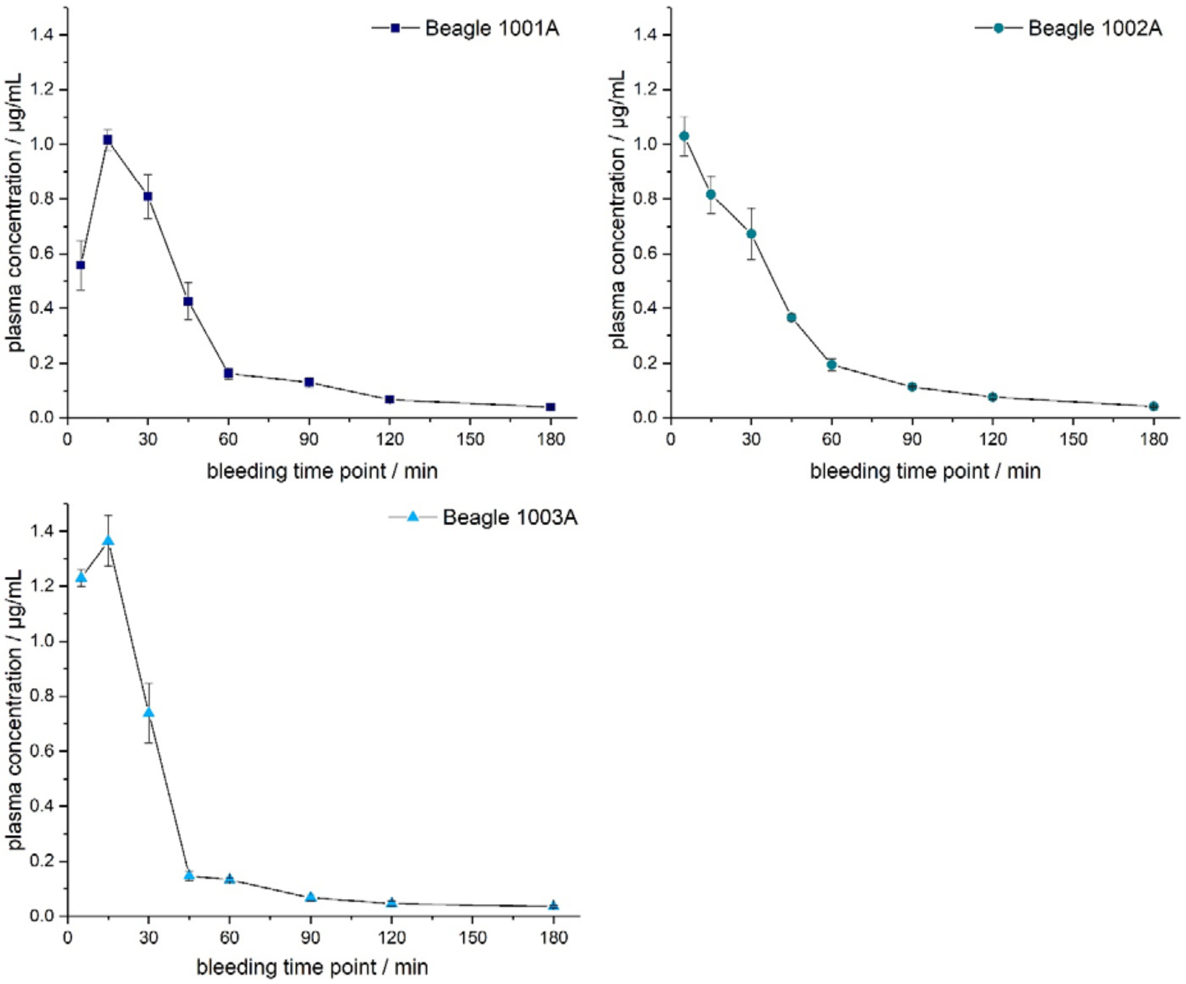

Figure S7: Plasma profile of ARV-1502 after intramuscular administration in three male beagle dogs (1001A to 1003A) at doses of 0.75 mg/kg body weight. Blood samples were collected in each animal after 5, 15, 30, 45, 60, 90, 120, and 180 min.
